# Pneumatosis intestinalis caused by *Cryptosporidium* colitis in a non-immunocompromised patient

**DOI:** 10.1016/j.idcr.2021.e01372

**Published:** 2021-12-22

**Authors:** Wesley Tang, Wamunyima Akakulu, Kunal Desai

**Affiliations:** a Department of Medicine, Kettering Medical Center, Kettering, OH, USA; b Clinical Assistant Professor, Department of Internal Medicine, Boonshoft School of Medicine, Wright State University, USA

**Keywords:** Cryptosporidium, Colitis, Pneumatosis intestinalis, Opportunistic infection

## Abstract

*Cryptosporidium* is an obligate enteric protozoan parasite commonly associated with severe symptoms such as profound diarrhea and dehydration in the immunocompromised, particularly those living with HIV/AIDS. In the immunocompetent, *Cryptosporidiosis* is self-limited, and characterized by mild non-bloody diarrhea with associated nausea and vomiting. We present an unusual case of presumed *Cryptosporidium* colitis, in an immunocompetent host, characterized by anorexia and pneumoperitoneum.

## Introduction

**Fig. 1:**
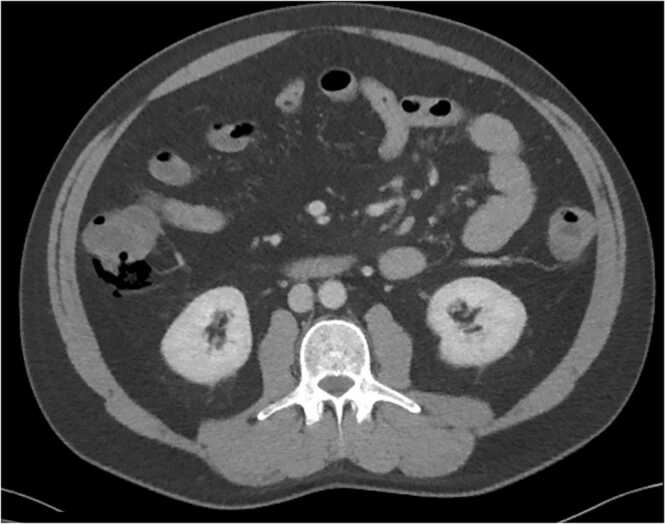
Free air and pneumatosis intestinalis adjacent to the descending colon/hepatic flexure, axial view.

**Fig. 2:**
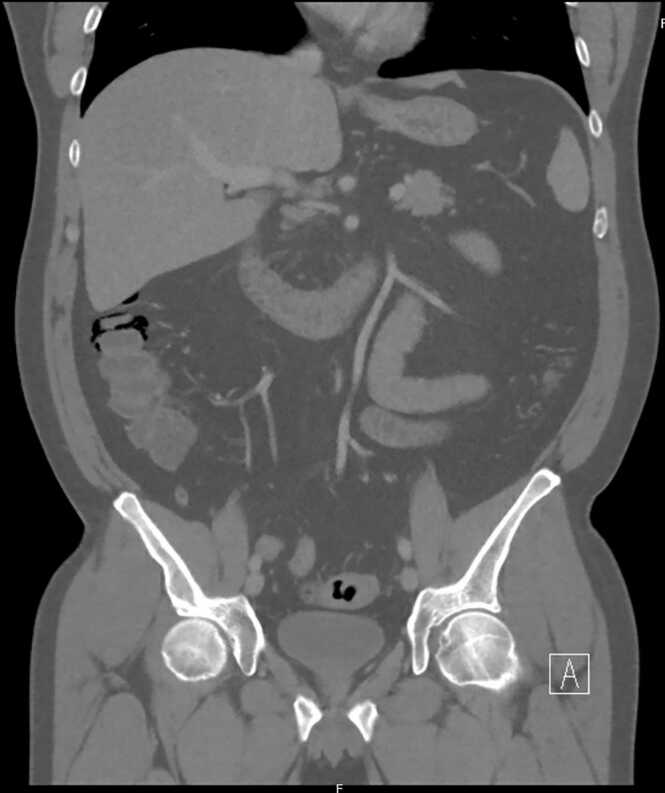
Free air and pneumatosis intestinalis adjacent to the descending colon/hepatic flexure, coronal view.

*Cryptosporidium* is an obligate enteric protozoan parasite whose association with gastrointestinal illness in humans was recognized in the late 1970 s [1]. The majority of human *Cryptosporidium* infections are attributed to *C. hominis* and *C. parvum* [2]*.* The parasite was established as an opportunistic pathogen associated with diarrhea in immunocompromised individuals [3]. By the 1980 s, *Cryptosporidium* infections became an acquired immunodeficiency syndrome (AIDS)-defining illness [4]. In immunocompetent hosts, cryptosporidiosis is typically asymptomatic or self-limited in adults. To the author’s knowledge, we present the first known case of pneumatosis intestinalis caused by presumed *Cryptosporidium* colitis, in a non-immunocompromised host. (Fig. 1, Fig. 2).

## Case description

A 49-year-old male cattle farmer with no significant medical or surgical history presented to the emergency department (ED) with 4 days of mild generalized abdominal pain, non-bloody diarrhea, weakness, and anorexia. The abdominal pain was characterized as cramps and rated at a 5 out of 10 in intensity. The diarrhea started gradually, did not appear to have any trigger, and persisted despite oral hydration with water and Pedialyte. Prior to symptom onset, the patient was reportedly in good health, not on any medications, and had no history of similar symptoms in the past. The patient did not report any nausea, vomiting, fevers, chills, melena, hematochezia, changes in diet, recent antibiotic use, recent travel, history of immunosuppression, or history of organ transplant. He also denied history of alcohol use, sick contacts, or unusual food ingestions. However, he did report that he had several newborn calves with diarrhea on his farm that he had been tending to recently.

When the patient arrived to the emergency department, he was afebrile and hemodynamically stable with a blood pressure of 117/87, pulse rate of 95 beats per minute, a temperature of 99.2 F and a respiration rate of 18. On physical exam, the patient was non-toxic, in mild distress with a soft, non-distended abdomen with no evidence of guarding or peritoneal signs. Laboratory workup was notable for a leukocytosis of 15,600 white blood cells (K/μL) with neutrophil predominance and mild elevation of monocytes. Basic metabolic panel revealed no metabolic acidosis and no kidney impairment with creatinine remaining stable at 1.1 mg/dL during the hospital stay. Transaminases were elevated at 484 and 1267 for aspartate aminotransferase (AST) and alanine aminotransferase (ALT) respectively— however the patient’s lipase, alkaline phosphatase, bilirubin, lactate, lactate dehydrogenase, and rapid testing for COVID-19 were within normal limits. Acetaminophen/paracetamol levels were undetectable. Computed tomography (CT) of the abdomen and pelvis with intravenous (IV) contrast revealed a small amount of free air and pneumatosis intestinalis adjacent to the ascending colon and hepatic flexure. After intravenous fluid hydration and empiric antibiotics with ertapenem, the patient was transferred to a larger referral hospital to be admitted under the general surgery department with a diagnosis of pneumoperitoneum with an associated pneumatosis of the hepatic flexure and transaminitis.

The general surgery team was consulted and antibiotics were switched to ciprofloxacin and metronidazole and additional testing was ordered including blood cultures, an acute viral hepatitis panel, stool pH and cultures, gastrointestinal antigen polymerase chain reaction (PCR) (BioFire® FilmArray® Gastrointestinal Panel), and *Clostridium difficile* testing. IV fluid hydration was continued, and gastroenterology was consulted due to the benign nature of the patient’s abdominal exam decreasing the likelihood of benefit from surgical intervention. The transaminitis was presumed to be shock liver secondary to dehydration from severe diarrhea which was further supported by the unremarkable acute viral hepatitis panel and the patient’s response to IV fluid hydration. Antinuclear antibodies, mitochondrial antibodies, and smooth muscle antibodies were ordered to rule-out autoimmune hepatitis and less likely primary biliary cirrhosis and primary sclerosing cholangitis – all of which resulted within normal limits. The GI (Gastrointestinal) PCR panel later returned positive for *Cryptosporidium* which prompted a consult to the infectious disease service who recommended the discontinuation of the previous antibiotics and the initiation of nitazoxanide 500 mg, every 12 h for a total of 6 doses. Stool studies were ordered, but never obtained. Human Immunodeficiency Virus (HIV) ELISA test was ordered and was found to be negative. After 12 h on nitazoxanide, the patient’s diarrhea significantly reduced in frequency. The patient was subsequently upgraded to a regular diet and was tolerated without issue. The pneumatosis and the pneumoperitoneum were presumed to be a result of the *Cryptosporidium* colitis as well given the absence of any other clear etiology. After a total of 3 days, the patient was discharged home in stable condition once his diarrhea had slowed down enough for the patient to maintain adequate hydration. Given that he completed a 3-day course of nitazoxanide while hospitalized, he was not discharged with further antimicrobial therapy. His leukocytosis had resolved and his AST/ALT downtrended to 74/356 U/L from a high of 484/1267 U/L upon presentation. The decision was made not to repeat imaging of the pneumoperitoneum with an improvement in symptoms and laboratory tests. He has not had a recurrence of symptoms since.

## Discussion

*Cryptosporidium* spp. are obligate, intestinal protozoan parasites transmitted via the fecal-oral route and are one of the most important waterborne pathogens worldwide [5]. Infections are most commonly seen developing countries with poor sanitation. *Cryptosporidium* spp. has been recognized as a significant threat to young children and immunocompromised patients with an estimate of nearly 500,000 deaths attributable in children under 5 years of age in developing countries in a study from 2015 [6]. Cryptosporidiosis is also well-recognized in patients living with acquired immune deficiency syndrome (AIDS) and those with solid organ transplants [7].

In immunocompetent hosts, *Cryptosporidiosis* and other intestinal parasitic infections are often self-limited. Typical symptoms include watery diarrhea, nausea, vomiting, and low-grade fever. In the immunocompromised, profuse diarrhea, primary acalculous cholecystitis, sclerosing cholangitis, and pancreatitis have been reported [8]. Traditionally, the parasite is identified via brightfield or fluorescent microscopy of stool specimens. Histopathological specimens, if obtained, can also diagnose the organism with hematoxylin and eosin staining. Increasingly, stool antigen PCR testing, enzyme-linked immunosorbent assay (ELISA), and immunochromatographic tests have overtaken microscopy [9].

At the time of this writing, nitazoxanide, is the only United States Food and Drug Administration (FDA) approved drug for the treatment of *Cryptosporidium*. It has activity against other protozoans as well such as Leishmaniasis, Giardiasis, and intestinal amebiasis [10]. Nitazoxanide has only been shown to improve the resolution of diarrhea, parasitological eradication, and improve mortality in HIV-seronegative patients, but not HIV-seropositive patients [11].

The authors concede that without microscopy and stool culture data, the possibility of active *Cryptosporidium* infection as the cause of this patient’s clinical presentation is somewhat weakened. We also concede that the GI PCR panel used to diagnose *Cryptosporidium* can also recognize non-viable organisms. As a result of these limitations, we present this case as that of presumed and active *Cryptosporidium* infection with subsequent colitis and pneumotosis.

The authors hypothesize that the source of the patient’s *Cryptosporidiosis* came from contamination of the man’s drinking water given that he reported several newborn calves with diarrhea on his farm. Interestingly, the degree of the patient’s illness far exceeded the patient’s expected clinical course. The patient reported no significant medical or surgical history, was not immunocompromised and did not report any other predisposing risk factors. He would require multiple days of hospitalization and was found to have complications of *Cryptosporidium* colitis such as pneumoperitoneum and pneumatosis intestinalis. The patient was successfully treated with nitazoxanide and has not had a recurrence of symptoms at follow-up.

The causative agent is often undetected in many gastroenteritis cases. We speculate that *Cryptosporidium* infections may be underdiagnosed and could be associated with serious complications such as this case. This case highlights the need to consider *Cryptosporidium* infection in the differential diagnosis as it may have greater pathogenic potential than previously recognized. Rapid identification will allow for more appropriate antimicrobial therapy.

## Ethical approval

The Kettering Health Network Institutional Review has determined this project does not meet the definition of human subject research according to federal regulations, #20201-038.

## Consent

Written informed consent was obtained from the patient for publication of this case report and accompanying images. A copy of the written consent is available for review by the Editor-in-Chief of this journal on request.

## Funding support

None.

## Author contributions statement

WT, WA, KD designed and conducted the research. KD provided the data. WT had primary responsibility for the final content. All authors have read and approved the final manuscript.

## Conflicts of interest statement

The authors declare that they do not have a conflict of interest.

## Code availability

Not applicable.
